# Data‐Driven SuStaIn Model of Disability Progression in Amyotrophic Lateral Sclerosis

**DOI:** 10.1002/acn3.70519

**Published:** 2026-08-30

**Authors:** Giammarco Milella, Veria Vacchiano, Vittorio Velucci, Stefano Zoccolella, Rocco Liguori, Giovanni Defazio

**Affiliations:** ^1^ Neurology and Stroke Unit “F.M. Puca” University Hospital AOU Consorziale Policlinico Bari Italy; ^2^ IRCCS Istituto delle Scienze Neurologiche di Bologna UOC Clinica Neurologica, Ospedale Bellaria Bologna Italy; ^3^ Department of Translational Biomedicine and Neuroscience Aldo Moro University of Bari Bari Italy; ^4^ IRCCS Neuromed Institute Pozzilli Italy

**Keywords:** clinical trials, functional phenotyping, latent variable modelling, motor neuron disease, SuStaIn

## Abstract

**Objective:**

To determine whether ordinal Subtype and Stage Inference (SuStaIn) applied to routine ALSFRS‐R item scores can identify reproducible disability progression patterns in amyotrophic lateral sclerosis (ALS) and provide clinically meaningful staging.

**Methods:**

We analysed baseline ALSFRS‐R item responses from 866 PRO‐ACT participants. Ordinal SuStaIn inferred subtype‐specific sequences of functional deterioration and assigned each participant to a subtype and a SuStaIn‐derived functional stage. Longitudinal stability was assessed across 3625 consecutive follow‐up visit pairs from 697 participants. Structural reproducibility was evaluated in an independent cohort of 301 consecutive ALS patients. Associations of baseline subtype and stage with survival and subsequent ALSFRS‐R decline were examined using Cox and piecewise‐linear models.

**Results:**

A three‐subtype solution identified fine motor‐, gross motor‐ and bulbar‐predominant patterns of early disability. Within each subtype, SuStaIn reconstructed ordered multidomain sequences of functional deterioration and assigned each participant a SuStaIn‐derived stage. Subtype assignment was stable across 90.4% of consecutive visit pairs, and stage was non‐decreasing in 98.8%. Subtype‐specific event ordering was reproduced in the validation cohort. Higher baseline stage was associated with increased mortality risk in the fine motor‐ and gross motor‐predominant subtypes, but not clearly in the bulbar‐predominant subtype. Baseline stage showed subtype‐dependent, non‐linear associations with subsequent functional decline, with acceleration up to mid‐stage breakpoints in the fine motor‐ and gross motor‐predominant subtypes and less evident stage‐dependent acceleration in the bulbar‐predominant subtype.

**Interpretation:**

Routine ALSFRS‐R item‐level data can define clinically interpretable ALS progression subtypes and latent SuStaIn‐derived functional stages. Joint subtype‐stage modelling may refine prognostic stratification and support prognosis‐informed trial enrichment.

## 1 Introduction

Amyotrophic lateral sclerosis (ALS) is a rapidly progressive neurodegenerative disease characterised by substantial clinical and prognostic heterogeneity [1, 2]. Age at onset, site of symptom onset, rate of functional decline and cognitive or behavioural involvement vary widely across patients, such that apparently similar cohorts may in fact contain multiple phenotypes following distinct trajectories [3]. Variability in the disease position at which patients are assessed represents a further source of heterogeneity: individuals with similar clinical phenotypes may be sampled at different points along the disease course and therefore exhibit different patterns of accumulated disability [4]. A framework that can jointly model the two dimensions of variability, namely differences in disease trajectory and differences in disease position at the time of assessment, may enable more granular patient stratification and improve the prediction of disease progression in both research and clinical settings [4, 5].

Subtype and Stage Inference (SuStaIn) is an unsupervised data‐driven framework that simultaneously identifies distinct disease subtypes and reconstructs their progression as ordered sequences of stages, each characterised by accumulating abnormalities. A key strength of the approach is that it can infer progression structure from cross‐sectional data, thereby separating heterogeneity in disease phenotype from heterogeneity in disease position [6, 7, 8]. SuStaIn has been used to characterise phenotypic and temporal heterogeneity across several neurological disorders, including Alzheimer's disease, frontotemporal dementia, Parkinson's disease and multiple sclerosis [6, 7, 8, 9]. More recently, it has also been applied to neuroimaging data from ALS–FTD spectrum [10]. However, its utility in ALS clinical stratification remains uncertain, particularly with respect to whether SuStaIn‐derived subtypes and stages based on routine clinical data can support prognosis and trial stratification.

To capture clinical heterogeneity in ALS, the SuStaIn algorithm needs to be applied to a large dataset and to refer to a widely used measure capable of disentangling clinical heterogeneity. The revised ALS Functional Rating Scale (ALSFRS‐R), a measure of functional disability routinely collected in both clinical practice and trials, offers such information as it spans bulbar, fine motor, gross motor and respiratory domains [11, 12]. Although the ALSFRS‐R summarises multidomain disability into a total score, it can also provide domain subscores that may differ substantially in patients with the same total score. The large number of items contributing to multidomain assessment (*n* = 12), together with the range of scoring in each item (0 = no function to 4 = full function) makes the ALSFRS‐R particularly suitable for exploring the clinical heterogeneity of the disease [11, 13]. Overall, the ALSFRS‐R provides an ordinal measure of functional disability that quantifies both the extent and distribution of impairment at the time of assessment. However, a single ALSFRS‐R assessment cannot establish the sequence in which individual items or domains previously deteriorated, nor the likely order of subsequent disability accumulation.

In the present study, we applied ordinal SuStaIn [14] to baseline ALSFRS‐R item scores from the Pooled Resource Open‐Access ALS Clinical Trials (PRO‐ACT) [15] repository to identify subtype‐specific pseudo‐longitudinal sequences of functional disability accumulation and derive a model‐inferred functional stage for each participant within the corresponding sequence.

## 2 Methods

### 2.1 Data Source and Inclusion Criteria

We performed a retrospective analysis of deidentified data from the PRO‐ACT repository [15], a large trial‐derived dataset of patients with ALS enrolled in completed clinical studies. Starting from the 11,675 participants included in the July 29, 2022 PRO‐ACT data release, we first selected 5874 participants who had an ALSFRS‐R assessment at baseline, defined as the first available assessment at trial entry. After excluding participants with a recorded score on item 5b (‘cutting food and handling utensils, with gastrostomy’) and retaining only those with complete data for the 12 ALSFRS‐R items used by SuStaIn, 3034 participants were identified. We then restricted the analytical cohort to the 866 participants who had complete baseline demographic and clinical data required for the present analyses, including age, sex, revised El Escorial diagnostic category [16], onset‐to‐diagnosis interval, and mortality/follow‐up information.

Baseline instrumental measures of respiratory function and muscle strength were not required as inclusion criteria, since they were not uniformly available across patients from contributing trials, and were therefore analysed only when recorded. Respiratory function was defined using the best available baseline percent‐predicted forced vital capacity (FVC). Muscle strength was summarised as a global MegaScore derived from hand‐held dynamometry, obtained by converting baseline measurements from the 12 assessed muscles into *z*‐scores using the reference mean and standard deviation reported by Shefner and colleagues [17], and then averaging these values across muscles. Baseline progression rate (ΔFS) 18 was calculated as (48—baseline ALSFRS‐R total score) divided by months from symptom onset to baseline [18].

Among the selected patients, we also extracted data (including total ALSFRS‐R score and domain subscores as well as vital status [death: yes/no]) from all available post‐baseline assessments without imposing a fixed follow‐up schedule. Participants contributed data up to the last recorded measurement available.

### 2.2 Subtype and Stage Inference

SuStaIn was applied to baseline ALSFRS‐R item‐score profiles. Because participants entered PRO‐ACT at different points along the disease course, their cross‐sectional baseline assessments were treated as snapshots sampled from different, initially unknown positions along functional progression [6]. Ordinal SuStaIn combines the distribution of these population‐level snapshots to infer subtype‐specific pseudo‐longitudinal sequences of functional deterioration and to estimate the position of each individual within each sequence [14]. Progression was represented as a series of one‐point transitions between adjacent ALSFRS‐R item scores (4 → 3 → 2 → 1 → 0). Given 12 ALSFRS‐R items, this yielded 48 ordered events, with each event corresponding to a one‐point worsening in a single item. Higher SuStaIn stages therefore reflected progressively lower ALSFRS‐R item scores. To account for measurement noise and occasional category misclassification, the probability assigned to the observed category was fixed at 0.90, with the remaining probability mass distributed uniformly across the alternative categories [14]. For each participant, the model returned posterior probabilities of subtype membership and an estimated functional stage.

#### 2.2.1 Model Fitting, Cross‐Validation and Selection of the Number of Subtypes

Models were fitted in pySuStaIn using the ordinal likelihood with multiple random start points (N_startpoints = 25) and MCMC sampling (N_iterations_MCMC = 250,000). We computed models with increasing numbers of subtypes up to a prespecified maximum (N_S_max = 6) using participant‐level *k*‐fold cross‐validation (*k* = 10; shuffled splits with a fixed random seed). Models were trained on *k*−1 folds and evaluated on held‐out folds using out‐of‐sample log‐likelihood and the cross‐validation information criterion (CVIC), as recommended in pySuStaIn guidance [6].

A pragmatic model‐selection approach was adopted, jointly considering out‐of‐sample fit and the marginal gains in CVIC with additional subtypes. The final solution was chosen at the point where further decreases in CVIC became modest (i.e., an elbow in the CVIC curve), while preserving clinical interpretability and stability of the inferred sequences as assessed using positional variance diagrams [6, 19]. The chosen model was then used to derive baseline subtype and stage assignments and, subsequently, to generate visit‐level assignments when applied to follow‐up ALSFRS‐R assessments for longitudinal stability analyses [6, 20]. Unless stated otherwise, inferential analyses focused on participants with confident subtype assignment, defined a priori as a posterior probability for the most likely subtype ≥ 0.50.

#### 2.2.2 Longitudinal Stability of Subtype/Stage Assignment

The SuStaIn model fitted using baseline ALSFRS‐R data alone was then applied to each available follow‐up ALSFRS‐R assessment, yielding visit‐level subtype membership probabilities and a SuStaIn‐derived functional stage at each timepoint [6, 19]. When duplicate records were present at the same timepoint, we retained the assessment with the highest posterior assignment probability. Subtype stability across consecutive visits was summarised descriptively and visualised using Sankey diagrams, while stage change over time was assessed by comparing inferred stage between visits *t* and *t* + 1.

#### 2.2.3 External Validation of Subtype‐Specific Progression Patterns

The SuStaIn algorithm was independently applied to 301 consecutive ALS patients recruited at the tertiary Motor Neuron Disease Centre of the University of Bologna, Italy between January 2010 and July 2025 (external validation cohort). To assess structural reproducibility, we compared the mean event position of each ALSFRS‐R item transition within aligned subtype‐specific sequences between the discovery and the validation cohorts. Agreement was quantified using Spearman's rank correlation coefficient (*ρ*), Lin's concordance correlation coefficient (CCC) and error metrics, namely mean absolute deviation (MAD) and root mean squared error (RMSE).

### 2.3 Statistical Analysis

Continuous variables were reported as median (interquartile range, IQR) and categorical variables as number (percentage). Statistical significance was defined as a two‐sided α level of 0.05.

Baseline differences across SuStaIn‐derived subtypes were assessed using Kruskal–Wallis tests for continuous variables and *χ* ^2^ tests for categorical variables, as appropriate. To examine the association of baseline clinical variables with SuStaIn stage and subtype, we computed regression models including stage, subtype and their interaction. For continuous outcomes, we used ordinary least‐squares models of the form *y* ~ stage × subtype. Global evidence for a stage effect and for a stage‐by‐subtype interaction was evaluated using Type II ANOVA, and subtype‐specific stage slopes with 95% confidence intervals were derived from the fitted interaction models. For revised El Escorial diagnostic category, we fitted multinomial logistic regression models including stage, subtype and their interaction; global significance was assessed using likelihood‐ratio tests comparing nested models. *p* values for the stage main effect and for the stage‐by‐subtype interaction were adjusted separately using the Benjamini–Hochberg false discovery rate procedure.

#### 2.3.1 Survival Analysis

Survival time was defined as the interval from baseline to death or, for censored observations, to the last available follow‐up. To examine the relationship of baseline SuStaIn subtype and stage to survival, we fitted subtype‐specific Cox proportional hazards models including baseline SuStaIn stage as a continuous predictor and estimated the hazard ratio associated with one‐stage increase. To assess whether the association between subtype and survival varied across the progression sequence, we stratified participants by tertiles of baseline SuStaIn stage and fitted Cox proportional hazards models within each tertile.

#### 2.3.2 Baseline Stage and Longitudinal Decline Speed

We evaluated whether baseline SuStaIn stage was associated with the subsequent rate of functional decline by estimating participant‐specific rates of change in ALSFRS‐R total score and examining their relationship with baseline SuStaIn stage, both in the full analytical cohort and within each subtype. Decline speed in ALSFRS‐R total score (points/month) was estimated from linear mixed‐effects models (with higher values indicating faster deterioration), participant‐specific random intercepts and participant‐specific random slopes. Fixed effects comprised time, baseline subtype and a time‐by‐subtype interaction to allow subtype‐specific average slopes. Participant‐specific slopes were extracted as points/month loss of ALSFRS‐R scores, and decline speed was defined as the negative slope so that higher values reflect faster deterioration. Analyses were restricted to the first 24 months of follow‐up and to participants with at least three visits. Associations between baseline stage and decline speed were then modelled using piecewise‐linear (‘hinge’) regression with a single breakpoint identified by grid search; 95% confidence intervals for fitted curves and breakpoint estimates were obtained by non‐parametric bootstrap.

#### 2.3.3 Incremental Prognostic Value of the SuStaIn Model Beyond Conventional ALSFRS‐R Measures

To determine whether SuStaIn subtype and functional stage provided incremental prognostic information beyond conventional baseline ALSFRS‐R measures, we modelled the participant‐specific ALSFRS‐R total decline rate over 0–24 months as the outcome. Analyses included participants with at least three longitudinal ALSFRS‐R assessments and complete predictor data. Two models were computed: the first model included age, sex, baseline ALSFRS‐R progression rate, onset‐to‐diagnosis interval and the four ALSFRS‐R domain subscores; the second model included the same clinical covariates and all 12 baseline ALSFRS‐R item scores. For each predictor set, an incremental model additionally included baseline SuStaIn subtype as a categorical variable and SuStaIn‐derived functional stage as a continuous variable. Nested models were compared using *R* ^2^, adjusted *R* ^2^, AIC and a partial *F*‐test assessing the joint contribution of subtype and stage. Out‐of‐sample performance was evaluated using 5‐fold cross‐validation at the participant level. Models were fitted in four folds and used to predict decline in the held‐out fold; pooled held‐out predictions were used to calculate cross‐validated *Q* ^2^.

### 2.4 Standard Protocol Approvals, Registrations and Patient Consents

The protocol, patient information, consent form and other relevant study documentation were approved by the ethics committee of the coordinating centre before study initiation [Local Independent Ethics Committee of the Regional University Hospital Polyclinic of Bari, Italy (n. 6778)]. The studies were performed in accordance with the Declaration of Helsinki and consistent with Good Clinical Practice. Before enrollment, all patients provided written informed consent.

## 3 Results

### 3.1 Study Populations

The demographic and clinical features of the PRO‐ACT cohort (*n* = 866) and the validation cohort (*n* = 301) are in Table 1. Compared with PRO‐ACT, the validation cohort was older at baseline and symptom onset, included a higher proportion of women and patients classified as possible ALS, had a longer onset‐to‐diagnosis interval, and showed differences in ALSFRS‐R total score as well as in bulbar and limb domain subscores; by contrast, the two cohorts did not differ in respiratory subscore (Table 1).

**TABLE 1:** Demographic and clinical characteristics of the PRO‐ACT cohort and validation cohort.

Variable	PRO‐ACT (*n* = 866)	Validation cohort (*n* = 301)	*p*
Participants, *n*	866	301	
Age at baseline, years	58 (50–65)	71.4 (63.9–77.4)	< 0.00001
Sex			
Female	317 (36.6%)	139 (46.2%)	0.00336
Male	549 (63.4%)	162 (53.8%)	
Revised El Escorial category			
Definite	420 (48.5%)	55 (18.3%)	< 0.00001
Probable	315 (36.4%)	121 (40.2%)	
Probable laboratory‐supported	107 (12.4%)	56 (18.6%)	
Possible	24 (2.8%)	69 (22.9%)	
Age at onset, years	56 (48–63)	69 (61–75)	< 0.00001
Onset‐to‐diagnosis interval, months	9.0 (5.9–13.4)	11.0 (6.0–21.0)	< 0.00001
ALSFRS‐R total score	37 (33–41)	39 (33–43)	0.00862
ALSFRS‐R domain subscores			
Bulbar (Q1–Q3)	11 (9–12)	10 (8–12)	0.00004
Fine motor (Q4–Q6)	8 (6–10)	9 (7–11)	0.00044
Gross motor (Q7–Q9)	7 (6–10)	8 (6–12)	0.00014
Respiratory (Q10–Q12)	12 (11–12)	12 (11–12)	0.9724

### 3.2 Model Selection and Subtype Solution

As prespecified in the Methods, cross‐validation metrics guided the selection of the primary subtype solution.

Although held‐out fit and CVIC continued to improve up to six subtypes, gains beyond three subtypes became progressively smaller, while model complexity doubled from 144 to 288 subtype‐specific event‐order positions. Increasing the number of subtypes was also associated with lower assignment certainty, with the median maximum posterior probability decreasing from 0.979 for three subtypes to 0.941 for six subtypes, and with the emergence of smaller, less clearly differentiated subgroups (Tables S1 and S2; Figure S1). The three‐subtype solution was therefore retained as the most parsimonious balance between out‐of‐sample fit, model complexity and assignment certainty. No participant was assigned to stage 38 or higher.

**FIGURE 1:**
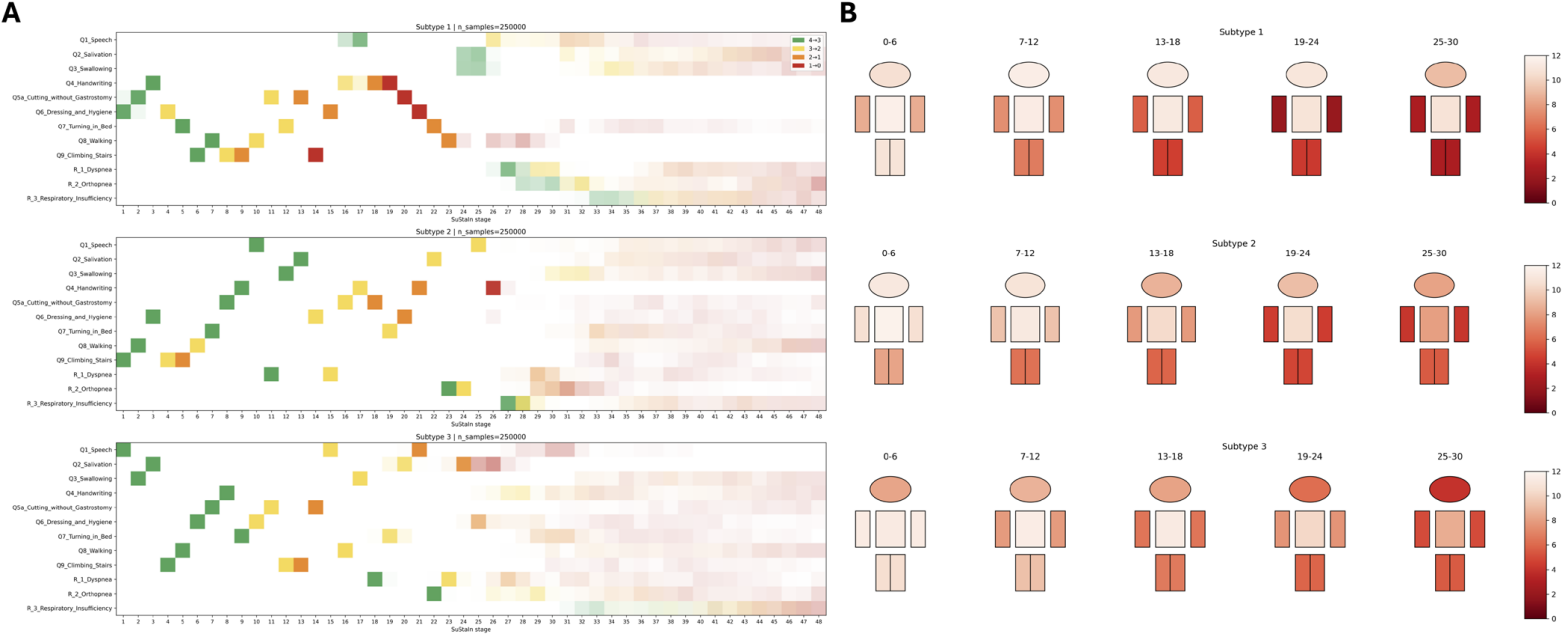
Subtype‐specific patterns of functional involvement across SuStaIn stage. (A) Sequence of ALSFRS‐R item involvement across SuStaIn stages for each inferred subtype. The x‐axis shows the progression of SuStaIn stages, with: Coloured tiles indicating the involvement of each ALSFRS‐R item and its one‐point transitions within that subtype (green = 4 → 3, orange = 3 → 2, yellow = 2 → 1, red = 1 → 0). (B) Mean ALSFRS‐R domain subscores (bulbar domain = head; fine motor domain = upper limb; gross motor domain = lower limb; respiratory domain = thorax) within each subtype, summarised across SuStaIn stage bands (0–6, 7–12, 13–18, 19–24, 25–30). White to red colour scale reflects decreasing mean domain subscore and worsening function.

The three data‐driven subtypes differed primarily in the functional domain dominating the earliest SuStaIn‐derived stages (approximately stages 1–10) (Figure 1): subtype 1 (S1, fine motor–predominant subtype) was characterised by early deterioration in handwriting, cutting food and handling utensils and dressing and hygiene; subtype 2 (S2, gross motor–predominant subtype) was characterised by early deterioration in walking, climbing stairs and rising from a chair; subtype 3 (S3, bulbar‐predominant subtype) was characterised by early deterioration in speech, salivation and swallowing. Regardless of the early differences in ALSFRS‐R domain subscores, the three subtypes were comparable for age, sex, revised El Escorial diagnostic category, ΔFS and percent‐predicted FVC, whereas MegaScore was lower in S1 (Table S3).

Beyond early subtype‐defining features (stage 1–10), the inferred subtype sequences showed an ordered multidomain spread across intermediate and later stages (Figure 1). In S1, early fine motor impairment was followed by involvement of bed mobility and lower‐limb items across roughly stages 10–20, whereas bulbar involvement emerged later, approximately between stages 20 and 30. In S2, upper‐limb fine motor items became more prominent during stages 10–20, and bulbar involvement occurred mainly during intermediate–later stages (approximately stages 15–30). In S3, spread to gross and fine motor items occurred approximately across intermediate stages, roughly stages 10–30. In all three subtypes, respiratory items clustered later in the inferred stage sequence, most often beyond stage 30.

**FIGURE 2:**
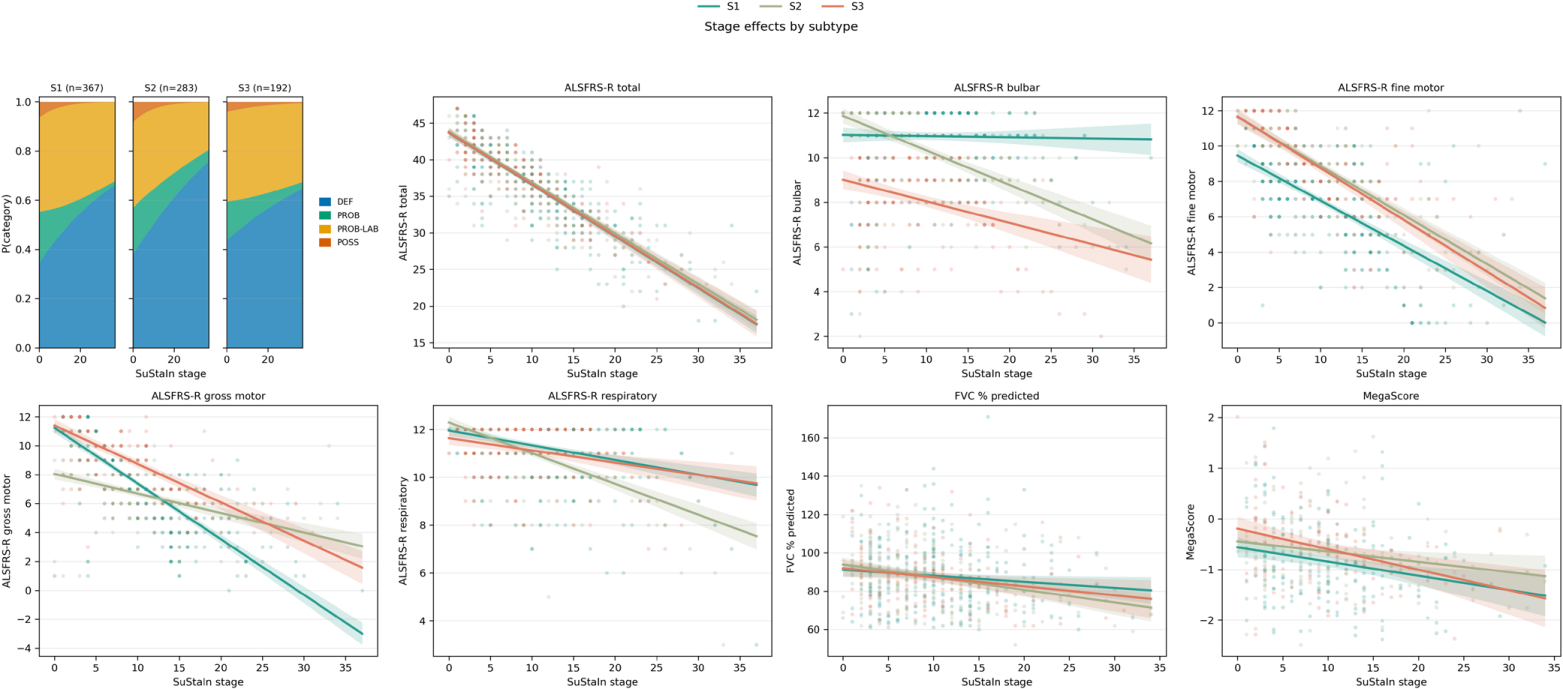
Diagnostic certainty and clinical severity trajectories across SuStaIn stages within each subtype. Panels show the association between SuStaIn stage and clinical measures after filtering to participants with subtype posterior probability ≥ 0.50. The left‐most panel displays the stage‐specific distribution of revised El Escorial diagnostic categories (stacked probabilities). The remaining panels show observed values (points) and model‐based fitted relationships (lines) between stage and ALSFRS‐R total score, ALSFRS‐R domain subscores (bulbar, fine motor, gross motor, respiratory), percent‐predicted FVC and MegaScore. Shaded bands indicate 95% confidence intervals.

Across all three subtypes, SuStaIn stage was associated with progressively lower baseline ALSFRS‐R total score, worse physiological measures, and greater revised El Escorial diagnostic certainty at higher stages, thus behaving as a coherent index of disease position (Figure 2, Table S4). By contrast, the association of SuStaIn stage with ALSFRS‐R domain subscores was subtype‐dependent and mirrored the subtype‐specific ordering of functional involvement: bulbar decline per stage was minimal in S1, more pronounced in S2 and S3; fine motor decline per stage was similar across subtypes; gross motor decline was steeper in S1 and S3 than in S2; and respiratory decline was greater in S2 (Figure 2, Table S4).

### 3.3 Longitudinal Stability of Baseline Subtype/Stage Assignment

**FIGURE 3:**
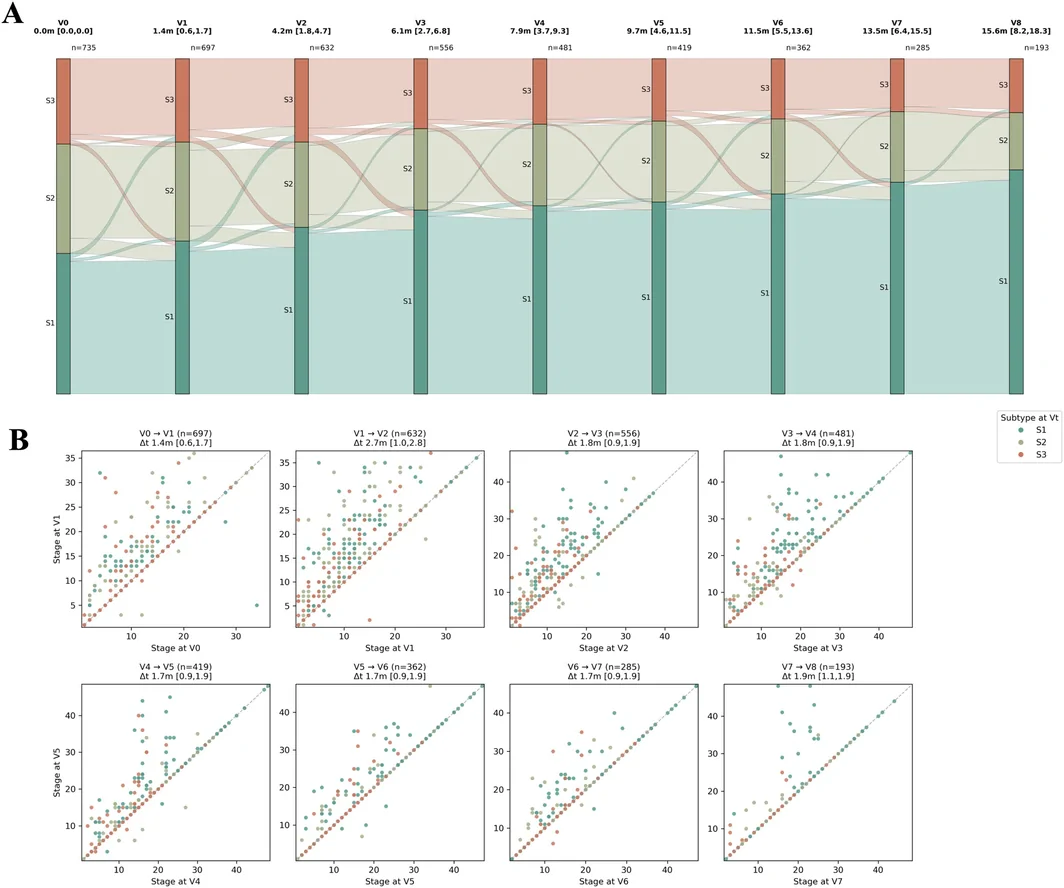
Longitudinal stability of SuStaIn subtype assignment and coherence of stage across consecutive visits. (A) Sankey diagram showing subtype transitions across consecutive follow‐up visits. Column headers report the median time since baseline and IQR for each visit (months), with n indicating the number of participants contributing to that visit. Bar height reflects the proportion of participants assigned to each subtype at that visit; connecting bands represent transitions between consecutive visits. (B) Scatter plots of SuStaIn stage at consecutive visits: points are coloured by subtype at visit t; the dashed diagonal denotes stage invariance, with points above/below the diagonal indicating stage increase/decrease between consecutive visits. Panel titles report the number of consecutive visit pairs and the median inter‐visit interval (Δt with IQR) in months.

Longitudinal ALSFRS‐R assessments yielded 3625 consecutive visit pairs from 697 participants, with a median inter‐visit interval of 1.7 months (IQR 0.9–2.0) (Figure 3A). When the fitted model was applied to longitudinal data, subtype assignment was largely stable across consecutive evaluations: 3277 of 3625 visit pairs (90.4%) retained the same subtype, whereas 348 (9.6%) involved subtype switching. Stability varied across subtypes, being highest in S1 (95.9%), lower in S3 (86.7%) and lowest in S2 (82.8%). The most frequent transition was from S2 to S1 (129 of 348 switches, 37.1%) (Figure 3A).

Stage assignment was even more coherent over time (Figure 3B). Across 3625 consecutive visit pairs, 3583 (98.8%) showed a non‐decreasing stage (Figure 3B). Median stage increased from 10 (IQR 6–14) at baseline to 17 (IQR 11–26) at the last available visit, corresponding to a median within‐participant increase of 6 stages (IQR 0–13) over a median cumulative follow‐up of 7.0 months (IQR 5.5–14.5). The median individual annualised rate of stage increase was 7.2 stages per year (IQR 0–17.1).

### 3.4 External Validation of Subtype‐Specific Progression Patterns

**FIGURE 4:**
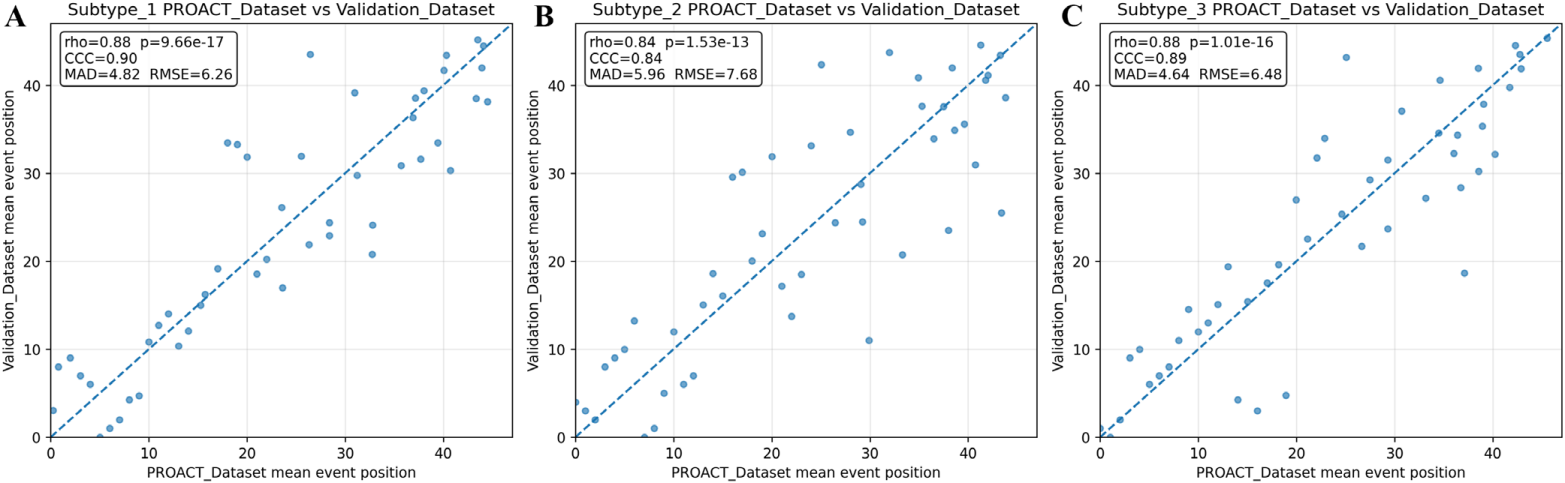
Concordance of mean event positions by subtype between PRO‐ACT and validation datasets. Scatter plots show the mean event position estimated in the PRO‐ACT dataset (*x*‐axis) versus an independent validation dataset (*y*‐axis) for (A) Subtype 1, (B) Subtype 2 and (C) Subtype 3. Each point represents a single event, and the dashed line indicates the line of identity (y = x). Agreement between datasets is summarised in each panel using Spearman's rank correlation (*ρ* and corresponding *p* value), Lin's concordance correlation coefficient (CCC), the median absolute deviation of the between‐dataset differences (MAD), and the root mean square error (RMSE).

We next assessed structural reproducibility by comparing the mean event position of each ALSFRS‐R item transition within aligned subtype‐specific sequences between the PRO‐ACT and validation datasets (Figure 4). Across all three subtypes, event ordering showed strong between‐cohort agreement, with points distributed close to the identity line. Concordance was high in S1 (Spearman's *ρ* = 0.88, *p* = 9.66 × 10^−17^; CCC = 0.90; MAD = 4.82; RMSE = 6.26), S2 (*ρ* = 0.84, *p* = 1.53 × 10^−13^; CCC = 0.84; MAD = 5.96; RMSE = 7.68) and S3 (*ρ* = 0.88, *p* = 1.01 × 10^−16^; CCC = 0.89; MAD = 4.64; RMSE = 6.48). Agreement was modestly lower in S2, indicating greater between‐cohort variability in the exact position of some events, but the overall subtype‐specific sequence structure remained well preserved.

### 3.5 Baseline SuStaIn Subtype/Stage and Survival

**FIGURE 5:**
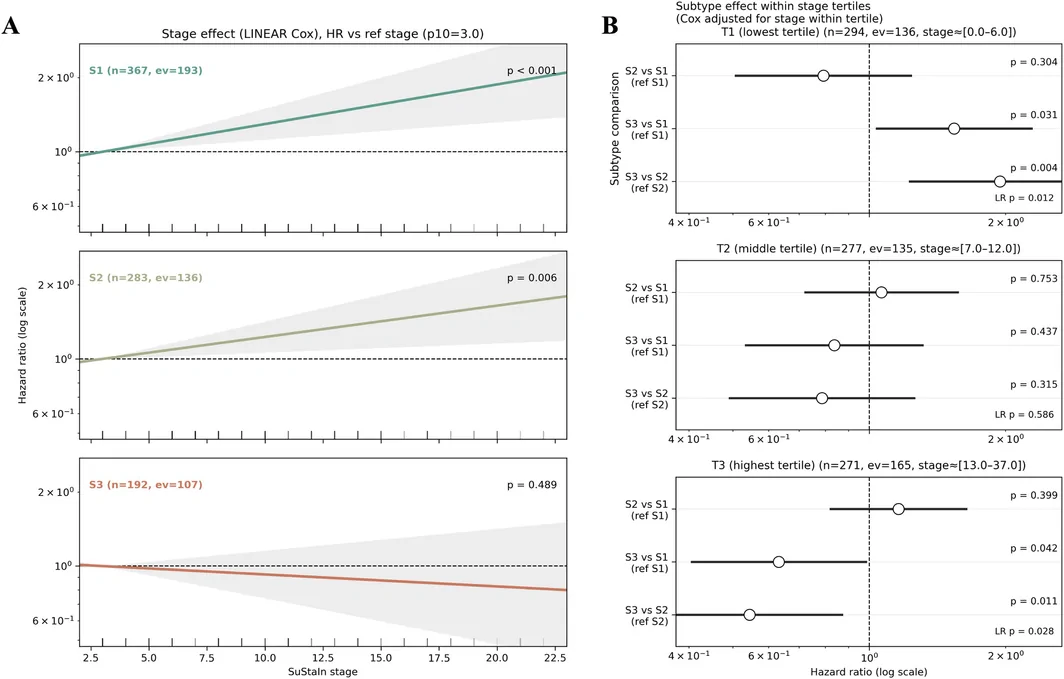
Survival analysis by SuStaIn‐derived subtype (subtype 1 = S1, subtype 2 = S2, subtype 3 = S3) and stage. (A) Stage effect within each subtype estimated by Cox proportional hazards models, with SuStaIn stage as a continuous predictor. Lines show the hazard ratio (HR) across the observed stage range using the 10th percentile (p10 = 3.0) as the reference stage (shaded bands denote 95% confidence intervals). The horizontal dashed line marks HR = 1. *p* values correspond to the Wald test for the stage coefficient. (B) Subtype effect within stage tertiles (T1–T3) estimated by Cox proportional hazards models including subtype and stage (centred within tertile) as covariates. Forest plots display subtype pairwise HRs on a logarithmic scale (points) with 95% confidence intervals (lines); the vertical dashed line marks HR = 1. *p* value from the likelihood‐ratio test assesses the incremental contribution of subtype over stage within each tertile.

In subtype‐specific Cox proportional hazards models including baseline stage as a continuous predictor (Figure 5A), each one‐stage increase was associated with a higher hazard of death in S1 (HR 1.04, 95% CI 1.02–1.06; *p* < 0.001) and S2 (HR 1.03, 95% CI 1.01–1.05; *p* = 0.006), whereas no significant association was observed in S3 (HR 0.99, 95% CI 0.96–1.02; *p* = 0.489).

We then examined whether subtype‐related differences in survival varied across the stage spectrum by stratifying participants into tertiles of baseline SuStaIn stage (T1: stages 0–6; T2: stages 7–12; T3: stages 13–37) and fitting Cox models within each tertile (Figure 5B). In the earliest tertile (T1), S3 showed a higher hazard of death than S1 and S2, whereas no difference was observed between S1 and S2; in the intermediate tertile (T2), hazard was similar across the three subtypes; and in the highest tertile (T3), S3 showed a lower hazard than S1 and S2, with no difference between S1 and S2.

### 3.6 Baseline SuStaIn Subtype/Stage and Rate of Functional Decline

Decline speed in ALSFRS‐R total score (points/month) over 0–24 months differed across subtypes, being highest in S1 and similar in S2 and S3: median decline speed was 1.15 (IQR 0.64–1.71), 0.91 (0.54–1.43) and 0.93 (0.60–1.39) points/month in S1, S2 and S3, respectively (*F* = 5.86, *p* = 0.003).

**FIGURE 6:**
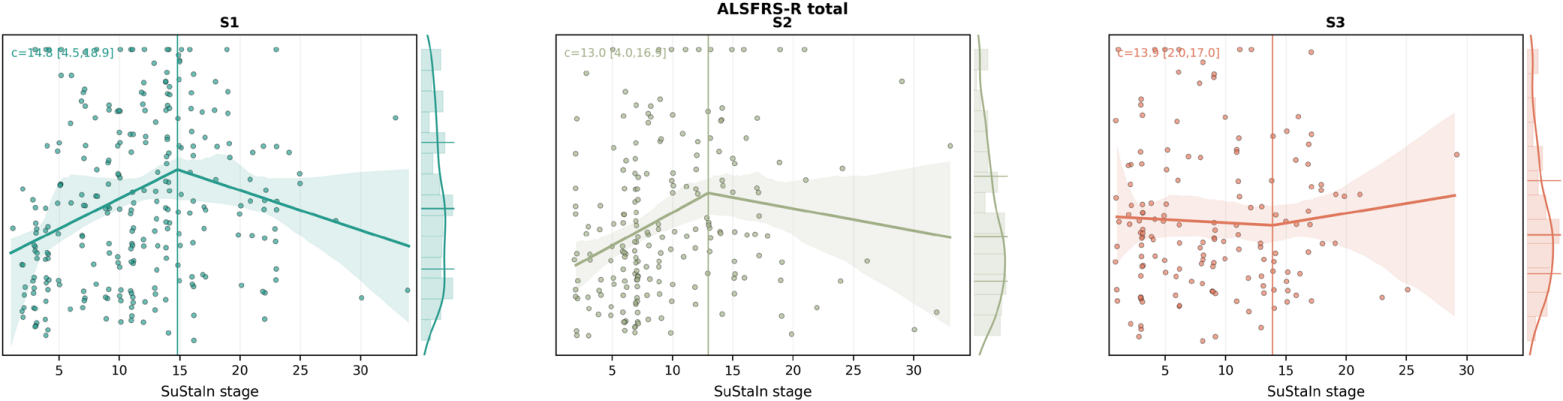
Baseline SuStaIn stage and subsequent ALSFRS‐R total decline speed over 0–24 months, stratified by subtype. Participant‐specific decline speed of ALSFRS‐R total score was estimated from linear mixed‐effects models with random intercepts and random slopes for time. Decline speed was defined as −slope (points/month), with higher values indicating faster deterioration. Each panel shows individual participant estimates plotted against baseline SuStaIn stage, with panels corresponding to subtypes 1–3 (S1–S3). Solid lines indicate subtype‐specific piecewise‐linear (‘hinge’) fits of decline speed versus baseline stage; shaded bands denote 95% bootstrap confidence intervals. The vertical line marks the estimated breakpoint, with its bootstrap uncertainty reported in the panel annotation. Right‐sided marginal histograms with overlaid kernel density curves summarise the distribution of decline speed within each subtype.

Baseline stage was associated with decline speed in a subtype‐dependent manner (Figure 6). In the overall sample, the relationship between stage and decline speed was best described by a hinge model with a late breakpoint at approximately stage 15. Similar stage breakpoints were observed in S1 and S2, with decline speed increasing across earlier stages and attenuating beyond the breakpoint (S1: stage 15, 95% CI 5–19; S2: stage 13, 95% CI 4–17). In contrast, S3 did not show the same progressive stage‐dependent acceleration: decline speed was already relatively high at earlier stages and did not increase consistently across subsequent stages (Figure 6).

### 3.7 Incremental Prognostic Value of the SuStaIn Model Beyond Conventional ALSFRS‐R Measures

Among 626 participants with longitudinal ALSFRS‐R follow‐up, SuStaIn subtype and functional stage provided incremental prognostic information about subsequent ALSFRS‐R decline. When subtype and stage were added to the model containing clinical covariates and ALSFRS‐R domain subscores, *R* ^2^ increased from 0.1866 to 0.2115 and adjusted *R* ^2^ from 0.1761 to 0.1973, while reduced AIC from 1138.6 to 1125.1 (partial *F*‐test, *p* = 2.6 × 10^−4^). Adding subtype and stage data to the second model including all 12 baseline ALSFRS‐R item scores, *R* ^2^ increased from 0.2071 to 0.2313 and adjusted *R* ^2^ from 0.1863 to 0.2072, while the AIC decreased from 1138.6 to 1125.2 (*p* = 3.0 × 10^−4^). Cross‐validated *Q* ^2^ increased from 0.155 to 0.168 in the domain‐based analysis and from 0.154 to 0.168 in the item‐level sensitivity analysis.

## 4 Discussion

In this cross‐sectional analysis of 866 participants from the PRO‐ACT cohort, ordinal SuStaIn applied to baseline ALSFRS‐R item‐score profiles identified three clinically interpretable trajectories of disability accumulation characterised by early fine motor, gross motor or bulbar predominance. Beyond assigning patients to subtypes, the model also reconstructed an ordered succession of disability positions within each subtype, with higher stages reflecting progressively greater global and domain‐specific impairment. This framework showed internal clinical coherence, retained substantial stability when projected onto longitudinal follow‐up data, and reproduced closely in an external validation cohort despite relevant demographic and clinical differences between the two populations.

SuStaIn disentangled two dimensions of ALS heterogeneity that are likely hidden by conventional ALSFRS‐R summary measures: the pattern through which disability accumulates (subtype) and the position of an individual patient along that pattern at the time of assessment (stage). Patients with the same ALSFRS‐R total score may carry very different configurations of impairment. As an illustrative example, two patients with an ALSFRS‐R total score of 36/48 may have different configurations of disability accumulation in our positional variance diagrams (Figure 1): one patient may show predominant fine motor impairment, with losses in handwriting, cutting food, dressing, or hygiene and relative preservation of bulbar and respiratory function, a profile that maps more closely onto the S1 trajectory; another patient may show prominent impairment in speech, salivation and swallowing, with relative preservation of limb and respiratory function, consistent with the early portion of the bulbar‐predominant S3 trajectory. Although the current distribution of functional impairment in an individual patient is directly observable in the ALSFRS‐R item profile, that profile alone does not indicate which functional transitions occurred earlier and which occurred later within the corresponding subtype‐specific trajectory. Obtaining this information directly would require longitudinal ALSFRS‐R assessments throughout the disease course. Rather than requiring observation of the same patient over the entire disease course, SuStaIn combines cross‐sectional profiles from patients sampled at different, initially unknown positions to estimate subtype‐specific pseudo‐temporal trajectories of functional deterioration [6, 14]. Knowing both the patient's subtype and stage would improve prognostic prediction beyond conventional ALSFRS‐R‐derived measures as suggested by our incremental sensitivity analysis.

Based on the aforementioned considerations, SuStaIn‐derived functional subtype and stage should be interpreted as statistical constructs rather than as a biologically validated model of ALS progression. Nevertheless, the subtype structure recovered by the model was clinically plausible. S1 and S2 broadly resembled spinal‐predominant forms, with early involvement of upper‐ and lower‐limb functional items, respectively, whereas S3 corresponded more closely to a bulbar‐predominant form. Even the succession of stages within each subtype was clinically plausible since ALS often begins focally and then spreads across anatomically and functionally connected systems [21, 22, 23]. Accordingly, in each subtype early stages were dominated by involvement of one functional territory, whereas intermediate and later stages showed broader multidomain spread and increasing overlap across subtypes, with respiratory items tending to occur later across all three trajectories [24].

The prognostic implications of subtype–stage assignment were further stressed by two additional observations. When survival was analysed according to baseline subtype and stage, mortality hazard increased progressively across higher stages in S1 and S2, suggesting that in these subtypes the risk of death accumulates as disability extends from the initially affected functional territory towards broader multidomain involvement. This pattern is consistent with what is usually observed in spinal‐predominant forms of ALS, in which mortality risk often reflects the convergence of progressive limb disability with respiratory insufficiency, impaired airway protection, dysphagia, malnutrition, aspiration and other complications that may emerge along the disease course [25, 26]. By contrast, mortality risk was less clearly dependent on reaching later stages in the S3 bulbar‐predominant trajectory. This finding is clinically plausible because bulbar dysfunction can affect survival earlier in the disease course through dysphagia, impaired airway protection, secretion burden, aspiration and nutritional compromise, even before widespread limb disability has accumulated [27, 28, 29, 30]. In this sense, subtype and stage should be interpreted jointly: later‐stage patients within S1 or S2 may be approaching mortality risk through progressive multidomain disability, whereas an earlier‐stage patient within a bulbar‐predominant trajectory may already carry clinically relevant risk because the functions affected first are directly linked to swallowing, nutrition, secretion management and airway safety.

A second prognostic implication of the SuStaIn modelling concerns the expected speed of functional decline after baseline assessment in overall functional status, as measured by changes in ALSFRS‐R score over follow‐up. ALS progression is commonly summarised using a single slope, such as ΔFS [18] or ALSFRS‐R decline over a fixed interval, under the assumption that past or average decline adequately represents the segment of disease that is still to come [31]. However, recent evidence indicates that ALSFRS‐R trajectories are often non‐linear, with progression rates varying across disease severity and over time [32, 33, 34, 35]. In our analysis, the fine motor‐ and gross motor‐predominant trajectories showed increasing ALSFRS‐R decline speed up to a mid‐stage breakpoint, followed by attenuation. This pattern is clinically plausible: when disease starts within a restricted functional territory, early ALSFRS‐R loss may be relatively contained; as impairment spreads into previously less affected domains, several items may begin to worsen in parallel, producing a faster observed decline. Once broader disability has accumulated, the slope may attenuate because fewer item scores retain substantial room to worsen and because rapidly deteriorating patients are more likely to be censored through death, clinical deterioration, or dropout. The bulbar‐predominant trajectory followed a different pattern. In S3, decline speed was already relatively high at earlier stages and showed limited additional acceleration as stage advanced. This should not be interpreted as evidence of a less active disease course. Rather, when bulbar dysfunction appears at the beginning of the trajectory, its consequences may extend beyond the bulbar ALSFRS‐R items themselves. Dysphagia, nutritional compromise, dehydration and increased respiratory burden can rapidly affect global function, vitality and daily performance, even before widespread limb disability has accumulated [28, 36, 37]. In this setting, clinically meaningful decline may be expressed early and may not require a later stage‐dependent acceleration to become prognostically relevant.

Information from SuStaIn modelling may be relevant not only for prognostic counselling but also for clinical trial design. ALS trials are still largely organised around broad eligibility criteria and baseline characterisation based on ALSFRS‐R total score, disease duration, site of onset, respiratory metrics and pre‐baseline progression estimate [38, 39]. These variables remain essential, but they do not fully distinguish patients who share similar global severity while differing in the configuration of disability and in their temporal position along the disease course. This clinical heterogeneity is not innocuous since it increases variance, reduces statistical power, and may dilute treatment effects when only a subset of enrolled participants is in the clinical and temporal window in which a therapeutic effect can be meaningfully expressed and detected [5, 40]. In this context, hidden heterogeneity in progression pattern (subtypes) and disease position (stages) should be made explicit before randomisation rather than handled only as residual noise after enrolment. Subtype‐stage inference may therefore provide a basis for stage‐restricted enrolment in time‐sensitive interventions, subtype‐stage‐stratified randomisation, or prespecified subgroup analyses aimed at distinguishing biological non‐response from treatment dilution in mixed populations.

This study has several strengths and limitations. PRO‐ACT is a trial‐derived resource and therefore reflects the selection filters of interventional research, with likely under‐representation of patients with very advanced disease and of those with substantial cognitive or behavioural involvement [15, 41]. However, successful application of the model in an external real‐world validation cohort suggests that the three‐subtype solution captures a robust clinical structure that can generalise across recruitment settings. In addition, the longitudinal stability of subtype assignment and the near‐monotonic behaviour of stage progression indicate that the framework is not simply identifying unstable cross‐sectional clusters, but clinically coherent trajectories that retain interpretability over follow‐up. We acknowledge that the observed subtype stability may partly reflect the persistence of the predominant clinical phenotype rather than providing independent validation of the latent progression model. It should be noted, however, that the same baseline subtype was retained by 90.4% of consecutive visit pairs, thus suggesting that the subtype generally remained recognisable despite the increasing multidomain disability and convergence as the disease progresses. The clinical plausibility of the subtype‐specific patterns of functional involvement also supports the relevance of subsequent analyses linking subtype and stage to survival and rate of functional decline. The model was built exclusively from ALSFRS‐R items and did not incorporate cognition, behaviour, upper and lower motor neuron burden, neurophysiology, imaging, genetics or fluid biomarkers. Ancillary measures were available only in subsets, follow‐up was irregular, and dropout was inevitably informative because of death or disease progression. In addition, SuStaIn infers ordered progression patterns from cross‐sectional item‐score configurations; therefore, the inferred sequences should be interpreted as data‐driven models of functional disability accumulation rather than direct reconstructions of biological spread.

Subtype‐stage modelling should not be considered a replacement for established staging or prognostic tools, but rather a complementary level of disease description. King's and MiToS stages index the accumulation of major clinical disability milestones [42, 43], ENCALS summarises overall survival risk from baseline clinical features and provides individualised estimates of prognosis [44], and blood or cerebrospinal fluid neurofilament light chain captures a biological dimension of neuroaxonal injury and adds prognostic value beyond clinical prediction models [45]. SuStaIn addresses different questions: (i) whether ALSFRS‐R item‐level configurations can be organised into reproducible subtype‐specific pseudo‐longitudinal sequences, and (ii) where an individual patient is positioned along one of these sequences.

## 5 Conclusions

Ordinal SuStaIn applied to baseline ALSFRS‐R item scores identified three clinically meaningful ALS subtypes, together with ordered within‐subtype stages that showed clinical coherence, longitudinal stability and external reproducibility. By jointly modelling subtype and stage, this approach addresses two major dimensions of ALS heterogeneity at baseline: the pattern of functional involvement and the position of the patient within the corresponding progression sequence. Subtype‐stage assignment may help refine prognosis in terms of survival and subsequent functional decline, while also informing trial enrichment, stratified randomisation and future biomarker‐integrated analyses. Rather than treating clinical heterogeneity as residual noise to be corrected after randomisation, subtype‐stage inference offers a way to explicitly describe clinical heterogeneity before enrollment and use this information in prognosis‐based analyses, study stratification and future biomarker integration.

## Author Contributions

**Giammarco Milella:** conceptualization, methodology, software, formal analysis, data curation, investigation, visualisation, project administration, writing: original draft, writing: review and editing. **Veria Vacchiano:** data curation, investigation, resources, validation, writing: review and editing. **Vittorio Velucci:** data curation, investigation, validation, writing – review and editing. **Stefano Zoccolella:** conceptualization, methodology, supervision, writing: review and editing. **Rocco Liguori:** resources, investigation, validation, supervision, writing: review and editing. **Giovanni Defazio:** conceptualization, methodology, supervision, project administration, writing: review and editing.

## Funding

The authors have nothing to report.

## Conflicts of Interest

The authors declare no conflicts of interest.

## Supporting information


**Figure S1:** Cross‐validation and assignment confidence for ordinal SuStaIn models fitted to individual ALSFRS‐R item scores in PRO‐ACT. (A) Distribution of held‐out test log‐likelihood per participant from *k*‐fold cross‐validation for models with increasing numbers of subtypes (N_S = 1–6): boxes show median and IQR; whiskers indicate 1.5 × IQR; points correspond to fold‐level estimates. (B) Cross‐validation information criterion (CVIC) as a function of N_S: lower values indicate better expected out‐of‐sample fit; the red marker denotes the minimum CVIC (N_S = 6). (C) Posterior probability of the most likely subtype (assignment confidence) by assigned SuStaIn stage for the three‐subtype solution (*n* = 854): boxes show median and IQR; the dashed horizontal line indicates the prespecified probability threshold (*p* = 0.50) used for high‐confidence assignments. (D) Distribution of assigned stages by subtype after applying the *p* ≥ 0.50 filter (*n* = 829), shown as stacked counts.
**Table S1:** Cross‐validation performance of ordinal SuStaIn models. Held‐out test log‐likelihood, cross‐validation information criterion and model complexity for solutions containing one to six subtypes.
**Table S2:** Posterior assignment certainty across SuStaIn solutions. Subtype sizes and distribution of maximum posterior assignment probabilities for models containing two to six subtypes.
**Table S3:** Baseline characteristics by SuStaIn subtype (S1–S3).
**Table S4:** Association between stage and baseline measures across subtypes computed by regression models including stage, subtype and their interaction. For continuous outcomes, results are reported as β coefficient (95% CI) per one‐stage increase. *p*‐interaction assesses differences in associations across subtypes. For revised El Escorial diagnostic categories, values are model‐predicted probabilities at the minimum and maximum observed stages with the corresponding change (Δ).

## Data Availability

The PRO‐ACT data that support the findings of this study are openly available through the Pooled Resource Open‐Access ALS Clinical Trials (PRO‐ACT) database. Data from the external validation cohort are available from the corresponding author upon reasonable request.
