# Supplementary material for: Data‐Driven SuStaIn Model of Disability Progression in Amyotrophic Lateral Sclerosis
Source: Ann Clin Transl Neurol. 2026 Aug 30:10.1002/acn3.70519. Online ahead of print. doi: 10.1002/acn3.70519 (PMC13527182; doi:10.1002/acn3.70519)
Supplement: Supplementary file 1 [file ACN3-9999-0-s001.docx]

**Supplementary Table 1. Cross-validation performance of ordinal SuStaIn models.**

Held-out test log-likelihood, cross-validation information criterion, and model complexity for solutions containing one to six subtypes.

| Number of subtypes | Subtype-specific event-order positions | PRO-ACT mean test log-likelihood (SE) | PRO-ACT CVIC |
| --- | --- | --- | --- |
| 1 | 48 | -16.563 (0.253) | 28,651.991 |
| 2 | 96 | -15.231 (0.240) | 26,275.040 |
| 3 | 144 | -14.620 (0.272) | 25,201.740 |
| 4 | 192 | -14.377 (0.282) | 24,761.885 |
| 5 | 240 | -14.229 (0.279) | 24,450.624 |
| 6 | 288 | -14.097 (0.275) | 24,243.848 |

**Supplementary Table 2. Posterior assignment certainty across SuStaIn solutions.**

Subtype sizes and distribution of maximum posterior assignment probabilities for models containing two to six subtypes.

| Number of subtypes | PRO-ACT subtype counts | Median maximum posterior probability | IQR | Posterior probability ≥0.75 | Posterior probability ≥0.90 | Posterior probability ≥0.95 |
| --- | --- | --- | --- | --- | --- | --- |
| 2 | 472 / 394 | 0.985 | 0.911-0.999 | 81.2% | 76.3% | 68.4% |
| 3 | 381 / 286 / 199 | 0.979 | 0.870-0.999 | 81.2% | 73.3% | 65.2% |
| 4 | 294 / 202 / 179 / 191 | 0.953 | 0.767-0.995 | 75.4% | 66.1% | 50.2% |
| 5 | 293 / 201 / 173 / 123 / 76 | 0.949 | 0.755-0.994 | 75.5% | 64.0% | 49.1% |
| 6 | 235 / 196 / 141 / 127 / 97 / 70 | 0.941 | 0.710-0.984 | 72.5% | 60.4% | 46.2% |

**Supplementary Figure 1: Cross-validation and assignment confidence for ordinal SuStaIn models fitted to individual ALSFRS-R item scores in PRO-ACT.**

(A) Distribution of held-out test log-likelihood per participant from k-fold cross-validation for models with increasing numbers of subtypes (N_S=1–6): boxes show median and IQR; whiskers indicate 1.5×IQR; points correspond to fold-level estimates. (B) Cross-validation information criterion (CVIC) as a function of N_S: lower values indicate better expected out-of-sample fit; the red marker denotes the minimum CVIC (N_S=6). (C) Posterior probability of the most likely subtype (assignment confidence) by assigned SuStaIn stage for the three-subtype solution (n=854): boxes show median and IQR; the dashed horizontal line indicates the prespecified probability threshold (p=0.50) used for high-confidence assignments. (D) Distribution of assigned stages by subtype after applying the p≥0.50 filter (n=829), shown as stacked counts.

**Supplementary Table 3.** Baseline characteristics by SuStaIn subtype (S1–S3).

|  |  | S1 | S2 | S3 | P value |
| --- | --- | --- | --- | --- | --- |
| Age, years |  | 57 (57–59) | 57 (57–61) | 57 (57–57) | 0.94 |
| Sex | Female | 121 (33.0%) | 110 (38.9%) | 80 (41.7%) | 0.91 |
|  | Male | 246 (67.0%) | 173 (61.1%) | 112 (58.3%) |  |
| El Escorial category | Definite | 169 (46.0%) | 141 (49.8%) | 96 (50.0%) | 0.50 |
|  | Probable | 43 (11.7%) | 41 (14.5%) | 21 (10.9%) |  |
|  | Probable laboratory supported | 146 (39.8%) | 91 (32.2%) | 70 (36.5%) |  |
|  | Possible | 9 (2.5%) | 10 (3.5%) | 5 (2.6%) |  |
| Onset-to-diagnosis interval, months |  | 8 (5–13) | 9 (6–14) | 9 (6–13) | 0.11 |
| ALSFRS-R total |  | 37 (33–40) | 38 (34–41) | 38 (34–41) | 0.02 |
| ALSFRS-R bulbar |  | 12 (11–12) | 11 (9–12) | 8 (7–10) | <0.01 |
| ALSFRS-R fine motor |  | 7 (5–9) | 10 (8–11) | 9 (7–12) | <0.01 |
| ALSFRS-R gross motor |  | 7 (5–10) | 6 (6–8) | 9 (7–11) | <0.01 |
| ALSFRS-R respiratory |  | 12 (11–12) | 12 (10–12) | 12 (11–12) | <0.01 |
| Progression rate |  | 0.67 (0.44–1.01) | 0.61 (0.39–1.01) | 0.67 (0.42–0.94) | 0.33 |
| Percent-predicted forced vital capacity |  | 85 (75.5–98) | 86.5 (75–98) | 87 (76–95) | 0.99 |
| MegaScore |  | -0.92 (-1.37–-0.45) | -0.70 (-1.24–-0.27) | -0.59 (-1.01–-0.17) | <0.01 |

**Supplementary Table 4.** Association between stage and baseline measures across subtypes computed by regression models including stage, subtype, and their interaction. For continuous outcomes, results are reported as β coefficient (95% CI) per one-stage increase. **P-interaction assesses** differences in associations across subtypes. For revised El Escorial diagnostic categories, values are model-predicted probabilities at the minimum and maximum observed stages with the corresponding change (Δ).

| Endpoint / category | Subtype 1  β coefficient (95% CI),  P value | Subtype 2  β coefficient (95% CI),  P value | Subtype 3  β coefficient (95% CI),  P value | p-interaction |
| --- | --- | --- | --- | --- |
| **ALSFRS-R total (point/stage)** | β = −0.71(−0.75, −0.66),  p<0.001 | β = −0.70(−0.74, −0.65),  p<0.001 | β = −0.71 (−0.77, −0.65),  p<0.001 | 0.929 |
| **ALSFRS-R bulbar (point/stage)** | β = −0.01(−0.03, 0.02),  p=0.683 | β = −0.15(−0.18, −0.13),  p<0.001 | β = −0.10 (−0.13, −0.06),  p<0.001 | <0.001 |
| **ALSFRS-R fine motor (point/stage)** | β = −0.26(−0.28, −0.23),  p<0.001 | β = −0.28(−0.31, −0.25),  p<0.001 | β = −0.29 (−0.33, −0.25),  p<0.001 | 0.308 |
| **ALSFRS-R gross motor (point/stage)** | β = −0.39(−0.41, −0.36),  p<0.001 | β = −0.14(−0.17, −0.10),  p<0.001 | β = −0.27 (−0.30, −0.23),  p<0.001 | <0.001 |
| **ALSFRS-R respiratory (slope/stage)** | β = −0.06(−0.08, −0.04),  p<0.001 | β = −0.13(−0.15, −0.11),  p<0.001 | β = −0.05 (−0.08, −0.03),  p<0.001 | <0.001 |
| **FVC % predicted, best (point/stage)** | β = −0.32(−0.60, −0.03),  p=0.029 | β = −0.66(−0.95, −0.37),  p<0.001 | β = −0.47 (−0.86, −0.08),  p=0.017 | 0.255 |
| **MegaScore (slope/stage)** | β = −0.03(−0.04, −0.01),  p<0.001 | β = −0.02(−0.04, 0.00),  p=0.016 | β = −0.04 (−0.06, −0.02),  p<0.001 | 0.345 |
| **El Escorial — Definite (prob min→max stage)** | 0.346→0.664  (Δ+0.318) | 0.380→0.759  (Δ+0.378) | 0.438→0.648  (Δ+0.210) | 0.948 |
| **El Escorial — Probable (prob min→max stage)** | 0.209→0.015  (Δ−0.193) | 0.190→0.047  (Δ−0.142) | 0.157→0.027  (Δ−0.130) | 0.948 |
| **El Escorial — Probable Lab-Supported (prob min→max stage)** | 0.383→0.320  (Δ−0.063) | 0.347→0.193  (Δ−0.154) | 0.364→0.320  (Δ−0.044) | 0.948 |
| **El Escorial — Possible (prob min→max stage)** | 0.063→0.001  (Δ−0.062) | 0.083→0.001  (Δ−0.082) | 0.040→0.005  (Δ−0.036) | 0.948 |
